# Teacher Awareness and Implementation of Food and Physical Activity Policies in Utah Elementary Schools, 2010

**Published:** 2011-12-15

**Authors:** William A. Lanier, Rachelle S. Wagstaff, Jessica H. DeMill, Michael D. Friedrichs, Julie Metos

**Affiliations:** Food and Drug Administration, Center for Food Safety and Applied Nutrition. At the time of the study, Dr Lanier was assigned to the Utah Department of Health as an Epidemic Intelligence Service (EIS) Officer, EIS Field Assignments Branch, Centers for Disease Control and Prevention. He is also affiliated with the US Public Health Service; Utah Department of Health, Salt Lake City, Utah; Utah Department of Health, Salt Lake City, Utah; Utah Department of Health, Salt Lake City, Utah; University of Utah, Salt Lake City, Utah

## Abstract

**Introduction:**

Schools are a key venue for childhood obesity prevention policies. The objective of this study was to examine factors associated with elementary school teacher awareness and implementation of their schools' food and physical activity policies.

**Methods:**

We collected data through an online survey of teachers at Utah elementary schools with food and physical activity policies. We used bivariate analysis and logistic regression to assess association of variables with teacher awareness and implementation.

**Results:**

Of 1,243 teacher respondents, 546 (44%) were aware of the food policy and 550 (44%) were aware of the physical activity policy. Food policy awareness was associated with knowing where written policies were located (odds ratio [OR], 2.7; 95% confidence interval [CI], 2.0-3.5), knowing the school health program coordinator (OR, 1.9; 95% CI, 1.4-2.7), and being reminded of policies at least once per semester (OR, 2.3; 95% CI, 1.7-2.9). Policy awareness was associated with both food (OR, 4.6; 95% CI, 3.6-6.0) and physical activity (OR, 1.6, 95% CI, 1.2-2.3) policy implementation. Helping develop the physical activity policy was associated with its implementation (OR, 2.4; 95% CI, 1.2-4.7). Thinking that students were more overweight than in the past was associated with food policy implementation (OR, 1.6; 95% CI, 1.1-2.5).

**Conclusion:**

Establishing food and physical activity policies at schools does not ensure teacher awareness or implementation. To promote policy awareness and implementation, school leaders should involve teachers in policy development, remind teachers of policies at least once per semester, and continue to educate teachers about childhood obesity.

## Introduction

Childhood obesity persists as a major public health problem (1-4). Legislators and researchers have called for the continued use of public policy to decrease the prevalence of childhood obesity (5,6). Schools are an ideal setting for obesity prevention policies because most children attend school (7), consume a large portion of their daily calories at school, and have opportunities at school for physical activity (8). Effective school health policies can improve student nutrition (9-12) and increase their participation in physical activity (11,13,14).

Studies have evaluated the extent to which nutrition and physical activity policies are established and implemented in schools and districts (9-21). Few studies have assessed awareness of policies as an outcome separate from implementation (17) or examined factors that influence policy implementation (9,13,16,18). One study noted that a lack of awareness among principals of district wellness policies impeded implementation (18), but no studies have examined teacher awareness of policies.

The Centers for Disease Control and Prevention (CDC) encourages state education and health agencies to develop coordinated school health programs to promote health-related policies (22). In 2001, the Utah Department of Health (UDOH) created the Gold Medal Schools (GMS) program (19,23) to promote student food and physical activity policies at elementary schools on the basis of CDC guidelines (24,25) and the Utah State Office of Education core curriculum (26).

By the end of the 2008-2009 school year, 206 (41%) of Utah's 507 public elementary schools were actively participating in the GMS program. GMS evaluation data collected in 2009, however, showed that only about half of teachers at GMS schools were aware of the GMS policies (UDOH, unpublished data, 2009). Increased teacher awareness and implementation of policies in Utah public elementary schools would likely increase policy effectiveness. The objective of this study was to examine factors associated with elementary school teacher awareness and implementation of food and physical activity policies at their schools.

## Methods

We conducted a cross-sectional study in April and May 2010 of the 4,380 regular classroom teachers at the 206 Utah public elementary schools (in 33 school districts) active in the GMS program. To participate and remain active in the GMS program, schools must establish and maintain the following 2 policies (among others) in written form: 1) food must not be used as a reward or punishment for student behavior and 2) students must participate weekly in a grade-specific minimum amount of structured physical activity (grades kindergarten through 2, ≥45 minutes; grades 3 through 6, ≥90 minutes). Each school in the GMS program identifies an in-school program coordinator who reports the status of policies at the school to UDOH and assists in creating, maintaining, and implementing policies.

We created a 27-question online survey (Appendix) by using Zoomerang (MarketTools, San Francisco, California). We adapted survey questions from those currently in use in GMS program performance measurement surveys. We also used findings from the literature and GMS teacher focus groups to guide question development. Before sending it to teachers, we administered the survey to 8 UDOH volunteers who were familiar with school health programs. We made minor changes to ensure it would be relevant, understandable, and easily navigated by teachers. The UDOH institutional review board approved the study, and a CDC human research protection coordinator also reviewed it. We calculated that at least 858 respondents would be necessary to accurately estimate teacher characteristics within 3%.

We asked teachers whether their school had the GMS food policy or GMS physical activity policy. Teachers who responded yes were considered to be aware of the policy; teachers who responded no or "don't know" were considered to be unaware of the policy. We asked teachers who indicated that they were aware of either the food or physical activity policy whether they helped develop each policy. We also asked teachers about implementation of the GMS food and physical activity policies, familiarity with the GMS program and coordinator, opinions about childhood obesity and its prevention, and their school's status for Adequate Yearly Progress (AYP), an academic performance measurement defined by the No Child Left Behind Act (27). We asked about certain characteristics of their class, their school, and themselves, including questions on the number of years they have been a teacher at their current school and at any school. For physical activity policy implementation, we asked teachers to identify a range of how many minutes of structured physical activity their students received each week. We did not include ranges of weekly physical activity less than 90 minutes as response options. (Because the GMS policy stipulates that students in grades kindergarten through 2 need only to receive a minimum of 45 minutes of weekly structured physical activity, we did not include teachers of these grades in the analysis of physical activity policy implementation.) To decrease potential social desirability bias and increase the response rate (by lowering the fear of negative repercussions), we did not collect any potentially identifying information about schools or teachers.

We gathered principal and teacher e-mail addresses from school Internet sites and by request to school districts. We asked principals by postcard and e-mail to forward the survey link to classroom teachers. We also sent teachers the survey link directly by e-mail. We informed teachers that survey participants would have the opportunity to be entered in a drawing for 1 of 200 online-redeemable $25.00 bookstore gift certificates. We stated on the e-mails and introductory survey page that teachers should take the survey only once. We closed the survey 1 month after launch. Teachers implied their consent to participate by advancing to each question on the survey. On the survey closing page, we invited teachers to submit their name via e-mail to the GMS program if they wished to be entered in the drawing.

After the close of the survey, we downloaded completed survey results as a Microsoft Excel (Microsoft Corporation, Redmond, Washington) spreadsheet. We performed all statistical analyses by using SAS version 9.2 (SAS Institute, Cary, North Carolina). For each variable, we excluded missing data from the analysis. We assessed factors for association with awareness and implementation of the food and physical activity policies in bivariate analysis. We used logistic regression to further assess association of variables significant in bivariate analysis. We calculated odds ratios (ORs) as measures of association and considered 95% confidence intervals (CIs) that excluded 1.0 to be significant. We assessed effect modification in logistic regression models by using interaction terms of relevant variables.

## Results

Of the 4,380 classroom elementary school teachers in the study, 1,243 (28%) responded to the survey. The percentage of respondents teaching in each grade was kindergarten, 9%; grade 1, 16%; grade 2, 16%; grade 3, 17%; grade 4, 16%; grade 5, 15%; grade 6, 12%.

Of all respondents, 546 (44%) were aware of the food policy at their school, 550 (44%) were aware of their school's physical activity policy, and 318 (26%) were aware of both policies. Among teachers aware of policies, 94 (17%) helped develop the food policy, and 80 (15%) helped develop the physical activity policy. Of all respondents, 404 (33%) reported that they never use food as a reward or punishment in their classroom; of these, 277 (69%) were aware of the food policy. Of the 710 teachers in grades 3 through 6, 333 (47%) were aware of their school's physical activity policy, and 275 (39%) reported that their students participated in 90 minutes or more of structured physical activity per week; of these, 160 (58%) were aware of the physical activity policy. Of the 710 teachers in grades 3 through 6, 88 (12%) reported implementation of both the food and physical activity policies.

Knowing the location of the school's written food and physical activity policies, being reminded of food and physical activity policies at least once per semester, and teaching at a school with a physical education (PE) specialist were each associated with both awareness and implementation of both food and physical activity policies (Table 1). No significant difference existed in awareness between teachers who reported being reminded once per semester versus once per month. Teacher sex, years teaching at the school, and number of students in class were not associated with awareness or implementation of either policy.

Teacher awareness of the food policy was associated with knowing the location of written policies, knowing the identity of the program coordinator, being reminded of policies at least once per semester, having a PE specialist at the school, and teaching at a school that failed AYP in the previous year (Table 2). Teacher implementation of the food policy was associated with being aware of the school's food policy and teaching at a school with 700 students or more. For physical activity policy awareness, a significant interaction existed between knowing the location of written policies and believing students to be more overweight than in the past. Knowing the location of written policies was significantly associated with awareness of the physical activity policy only among teachers who thought students were more overweight than they used to be. Other factors associated with awareness of the physical activity policy were knowledge of the program coordinator, being reminded of policies at least once per semester, having a PE specialist at the school, having more than 5 years of teaching experience, and thinking that the school offers adequate time for physical activity. Teacher-reported implementation of the physical activity policy was significantly associated with being reminded of policies at least once per semester, being aware of the school's physical activity policy, and thinking that the school offered adequate time for physical activity. Neither thinking the school had adequate physical activity facilities nor having faculty meetings at least weekly was associated with awareness or implementation in any of the models.

For teachers aware of the food policy, believing students to be more overweight than they used to be was significantly associated with food policy implementation in logistic regression (Table 3). For teachers aware of the physical activity policy, having a role in developing the policy, being reminded of the policy at least once per semester, and thinking that the school had adequate time for physical activity were associated with physical activity policy implementation in logistic regression.

## Discussion

Although Utah's GMS program has been successful in encouraging schools to establish childhood obesity prevention policies (28), less than half of the teachers at elementary schools active in the GMS program were aware of the food and physical activity policies, and a minority of teachers reported implementation of these policies. Clearly, school health programs must do more than establish policies.

For school policies to achieve their intended outcome, at least 3 actions are necessary: 1) policies should be established at schools, 2) school personnel should be made aware of the policies, and 3) policies should be implemented by school personnel. After a policy is established, multiple factors at the school can enhance or impede policy awareness and implementation. Our study demonstrates that teacher awareness of policies promotes policy implementation. We also identify simple interventions — such as involving teachers in policy development and keeping them continually apprised of school policies — to improve teacher awareness and implementation of policies.

Two survey findings highlight the need to educate teachers about the childhood obesity epidemic: 1) knowing the location of written policies was associated with physical activity policy awareness only among teachers who thought students were more overweight than they used to be (75% had this opinion); and 2) believing that students were more overweight than they used to be was associated with food policy implementation. Teachers who view childhood obesity as an emerging concern likely place more emphasis on policies intended to prevent obesity in children.

The finding that teachers were more likely to be aware of policies if their school had a PE specialist reinforces existing recommendations for schools to have a certified faculty member dedicated to PE (22). The finding that teaching at a school that failed AYP in the previous year was associated with teacher awareness of food policy was somewhat unexpected. One explanation could be that schools that pass AYP may prioritize academic subjects over childhood obesity prevention policies. Research (17,29) suggests that emphasis on health subjects in schools has decreased since the advent of academic testing mandated by No Child Left Behind (27). Additional factors significantly associated with teacher awareness and implementation of policies included a longer teaching career, larger school size, and thinking that a school had adequate time for student physical activity; these contribute to the understanding of factors that might influence policy effectiveness but do not lend themselves easily to intervention.

Research on factors that influence food and physical activity policy awareness and implementation at elementary schools, particularly among teachers, is lacking. This study identifies several such factors. Future research should focus on additional factors (eg, emphasis on health subjects, student socioeconomic status [15-17,30], school-area population density [15,30]). Our findings apply to Utah elementary schools active in the GMS program, but they may also apply to other elementary schools with childhood obesity prevention policies.

This study has several limitations. First, because we did not collect identifying information about schools, we could not control for school-level factors not included on the survey (eg, student socioeconomic status and school-area population density) or assess potential clustering effects based on different numbers of respondents per school. However, multivariate analysis of previous GMS survey data indicated that eligibility for free or reduced-price lunch and whether the school's county was urban or rural were not significantly associated with teacher awareness of the policies and that there was no significant clustering effect on the estimates by number of respondents per school (unpublished GMS survey data, 2008-2009). Second, although the anonymity of the survey might have encouraged more teachers to respond and to be more candid in their responses, it also prevented us from determining how nonrespondents differed from respondents, which is important, given the 28% response rate. Nonrespondents would arguably be less interested in GMS policies than respondents and be expected to report lower awareness and implementation of food policies. The purpose of this study, however, was to determine factors associated with policy awareness and implementation; it is unclear whether nonrespondents would differ significantly from respondents on these factors. Some evidence suggests that respondents were representative of the target teacher population. For example, the distribution of respondents among grade levels was similar to the distribution of the teachers in the population surveyed. In addition, we ascertained from the identified data collected in the gift certificate drawing, which 49% of respondents entered, that respondents represented at least 79% of the districts targeted by the survey. The third major limitation was that analysis of factors associated with implementation of the physical activity policy was limited to responses from teachers in grades 3 through 6, so findings on physical activity policy may not be generalizable to other grades.

We recommend that teachers be educated on the growing problem of childhood obesity and, if possible, be involved in the creation of obesity prevention policies. Once schools establish such policies, we further recommend that program coordinators and school leaders remind teachers at least once per semester about policy content and the location of written policies. Leaders of state education departments, coordinated school health program managers, school administrators, and others involved in childhood obesity prevention policies in elementary schools should consider applying the findings of this study to coordinated school health programs.

## Figures and Tables

**Table 1:** Factors Associated in Bivariate Analysis With Elementary School Teacher Awareness and Implementation of Food and Physical Activity Policies, Utah, 2010^a^

**Variable^b^**	Food Policy		Physical Activity Policy	

	Awareness	Implementation	Awareness	**Implementation** c
Knows location of written policies	+	+	+	+
Knows who the program coordinator is	+	+	+	−
Reminded of policies at least once per semester	+	+	+	+
Thinks students are more overweight than in the past	−	−	+	−
Aware of the food policy	NA	+	NA	NA
Aware of the physical activity policy	NA	NA	NA	+
School has physical education specialist	+	+	+	+
School failed Adequate Yearly Progress in the prior year	+	−	−	−
No. of students in school	−	+	−	−
Years in a teacher's career	−	−	+	−
Thinks school has adequate time for student physical activity	−	−	+	+
Thinks school has adequate facilities for student physical activity	−	−	+	+
School has faculty meetings at least weekly	+	−	+	−

Abbreviation: NA, not applicable to this analysis.

a Plus sign (+) indicates a significant positive association; minus sign (−), not a significant positive association.

b Evaluated variables found not to be significantly associated with awareness or implementation of either policy are not shown. They include teacher sex, years teaching at the school, and number of students in class.

c Analysis of physical activity policy implementation was restricted to teachers of grades 3-6.

**Table 2:** Logistic Regression of Factors Associated with Elementary School Teacher Awareness and Implementation of Food and Physical Activity Policies, Utah, 2010

Variable^a^	Food Policy		Physical Activity Policy	

	Awareness (n = 1,243)	Implementation (n = 1,242)	Awareness (n = 1,243)	**Implementation** b (n = 710)

	**OR** **(95% CI)** c			
**Knows location of written policies**	2.7 (2.0-3.5)	NS	NS	NS
Knows location of written policies (only among teachers who think students are more overweight than in the past)	NA	NA	2.5 (1.8-3.4)^d^	NA
Knows location of written policies (only among teachers who do not think students are more overweight than in the past)	NA	NA	1.1 (0.7-1.9)^d^	NA
**Knows who the program coordinator is**	1.9 (1.4-2.7)	NS	1.6 (1.2-2.3)	NI
**Reminded of policies at least once/semester**	2.3 (1.7-2.9)	NS	1.8 (1.4-2.4)	2.0 (1.4-2.7)
**Aware of the food policy**	NA	4.6 (3.6-6.0)	NA	NA
**Aware of the physical activity policy**	NA	NA	NA	1.6 (1.2-2.3)
**School has physical education specialist**	1.7 (1.3-2.2)	NS	1.7 (1.4-2.2)	NS
**School failed Adequately Yearly Progress in the prior year**	2.0 (1.3-3.0)	NI	NI	NI
**No. of students in school**				
<500	NI	1 [Reference]	NI	NI
500-699		1.0 (0.7-1.3)		
≥700		1.4 (1.02-2.0)		
**Years taught in career**				
1-4	NI	NI	1 [Reference]	NI
5-9			1.6 (1.1-2.4)	
10-19			1.7 (1.2-2.4)	
≥20			1.5 (1.03-2.1)	
**Thinks school has adequate time for student physical activity**	NI	NI	1.9 (1.4-2.4)	3.1 (2.2-4.3)

Abbreviations: OR, odds ratio; CI, confidence interval; NA, not applicable to this analysis; NI, not included in model because variable was not significant in bivariate analysis; NS, not significant in final versions of models.

a Evaluated variables found not to be significantly associated with awareness or implementation of either policy are not shown. They include thinking the school had adequate facilities for physical activity and having faculty meetings at least weekly.

b Analysis of physical activity policy implementation was restricted to teachers of grades 3-6.

c For dichotomous variables, the referent group is the opposite of the indicated category.

d Knowing where policies are posted was significantly associated with physical activity policy awareness only among teachers who thought students were more overweight than in the past (significant interaction term).

**Table 3:** Bivariate Analysis and Logistic Regression of Factors Associated With Elementary School Teacher Implementation of Food and Physical Activity Policies Among Teachers Aware of the Policies, Utah, 2010

Variable	Food Policy (n = 546)		Physical Activity Policy (n = 333)^a^	

	Bivariate	Multivariate	Bivariate	Multivariate

	OR (95% CI)^b^			
Helped develop policy	1.6 (1.02-2.5)	NS	2.9 (1.6-5.5)	2.4 (1.2-4.7)
Thinks students are more overweight than in the past	1.6 (1.1-2.4)	1.6 (1.1-2.5)	NS	NI
Reminded of policies at least once/semester	NS	NI	1.9 (1.3-3.0)	2.1 (1.3-3.4)
Thinks school has adequate time for student physical activity	NS	NI	3.2 (2.0-5.1)	3.1 (1.9-5.0)
Thinks school has adequate facilities for student physical activity	NS	NI	1.9 (1.2-3.1)	NS

Abbreviations: OR, odds ratio; CI, confidence interval; NI, not included in model because variable was not significant in bivariate analysis; NS, not significant in final versions of models, ORs not calculated.

a Analysis of physical activity policy implementation was restricted to teachers of grades 3-6.

b For all variables, the referent group is the opposite of the indicated category.

## Appendix. Survey Questions, Teacher Awareness and Implementation of Food and Physical Activity Policies in Utah Elementary Schools, 2010

**Page 1 - Question 1 - Choice - One Answer (Bullets)**

How often are faculty meetings held at your school?

- Daily
- Weekly
- Monthly
- Once per semester
- Once per year
- Never

**Page 1 - Question 2 - Choice - One Answer (Bullets)**

What position does your Gold Medal Schools coordinator hold?

- Classroom teacher
- Parent/PTA member
- Principal
- PE specialist
- Don't know
- My school used to have one, but not anymore
- Other, please specify

**Page 1 - Question 3 - Choice - One Answer (Bullets)**

Does your school have a PE specialist?

- Yes
- No
- Don't know

**Page 1 - Question 4 - Choice - One Answer (Drop Down)**

About how many minutes of structured physical activity do your students receive each week? (Includes gym time, in-class exercising, and organized games, but excludes recess.)

- Less than 90 minutes each week
- 90-119 minutes each week
- 120-149 minutes each week
- 150 minutes or more each week
- None

**Page 1 - Question 5 - Choice - One Answer (Bullets)**

How often do you use food as a reward or punishment for student behavior in your classroom?

- At least once a day
- At least once per week
- At least once per month
- Less than once per month
- Never

**Page 1 - Question 6 - Choice - Multiple Answers (Bullets)**

If applicable, for what reasons do you not use food as a reward or punishment for student behavior in the classroom? (You may choose more than one, if applicable.)

- School policy
- District policy
- School culture/tradition
- Using food as a reward or punishment is not a good idea
- Other, please specify

**Page 2 - Question 7 - Choice - One Answer (Bullets)**

What is your gender?

- Female
- Male

**Page 2 - Question 8 - Choice - Multiple Answers (Bullets)**

What grade(s) do you teach? (You may choose more than one, if applicable.)

- K
- 1
- 2
- 3
- 4
- 5
- 6
- I am not a classroom teacher

**Page 2 - Question 9 - Open Ended - One Line**

How many students are there in your class?

**Page 2 - Question 10 - Choice - One Answer (Drop Down)**

About how many students are there in your school?

- Less than 100
- 100-199
- 200-299
- 300-399
- 400-499
- 500-599
- 600-699
- 700-799
- 800 or more

**Page 2 - Question 11 - Open Ended - One Line**

How many years have you been teaching at this school?

**Page 2 - Question 12 - Open Ended - One Line**

How many years have you been teaching at any school?

**Page 2 - Question 13 - Choice - One Answer (Bullets)**

Did your school pass Adequate Yearly Progress (AYP) in school year 2008-2009 (last year)?

- Yes
- No
- Don't know

**Page 3 - Question 14 - Choice - One Answer (Bullets) [Mandatory]**

Does your school have a policy requiring students to receive a minimum number of minutes of structured physical activity? (Includes gym time, in-class exercising, and organized games, but excludes recess.)

- Yes [Skip to page 4]
- No [Skip to page 5]
- Don't know [Skip to page 5]

**Page 4 - Question 15 - Choice - Multiple Answers (Bullets)**

From whom did you learn about this physical activity policy at your school? (You may choose more than one, if applicable.)

- Another teacher
- Principal
- Gold Medal School coordinator
- PE specialist
- Other, please specify

**Page 4 - Question 16 - Choice - One Answer (Bullets)**

Were you involved in developing this physical activity policy at your school?

- Yes
- No

**Page 5 - Question 17 - Choice - One Answer (Bullets) [Mandatory]**

Does your school have a policy that food is not to be used as a reward or punishment in the classroom?

- Yes [Skip to page 6]
- No [Skip to page 7]
- Don't know [Skip to page 7]

**Page 6 - Question 18 - Choice - Multiple Answers (Bullets)**

From whom did you learn about this food policy at your school? (You may choose more than one, if applicable.)

- Another teacher
- Principal
- Gold Medal School coordinator
- PE specialist
- Other, please specify

**Page 6 - Question 19 - Choice - One Answer (Bullets)**

Were you involved in developing this food policy at your school?

- Yes
- No

**Page 7 - Question 20 - Choice - One Answer (Bullets)**

How often are you reminded of the physical activity and/or food policies at your school?

- Once per week
- Once per month
- Once per semester
- Once per school year
- Never
- My school doesn't have these policies

**Page 7 - Question 21 - Choice - Multiple Answers (Bullets)**

Where are your school's written policies about physical activity and/or food kept at your school? (You may choose more than one, if applicable.)

- In the principal's/administrators' office
- In the break room/teacher's lounge
- Posted on the school website
- These policies are not written at my school
- Don't know
- Other, please specify

**Page 8 - Question 22 - Choice - One Answer (Bullets)**

Do you think physically active students perform better academically than sedentary students?

- Yes
- No
- Don't know

**Page 8 - Question 23 - Choice - One Answer (Bullets)**

Do you think elementary students are more overweight than they used to be?

- Yes
- No
- Don't know

**Page 8 - Question 24 - Choice - One Answer (Bullets)**

Do you think appropriate nutrition in school is important for a child to maintain a healthy weight?

- Yes
- No
- Don't know

**Page 8 - Question 25 - Choice - One Answer (Bullets)**

Do you think adequate physical activity in school is important for a child to maintain a healthy weight?

- Yes
- No
- Don't know

**Page 8 - Question 26 - Choice - One Answer (Bullets)**

Do you think that facilities for student physical activity are adequate at your school?

- Yes
- No
- Don't know

**Page 8 - Question 27 - Choice - One Answer (Bullets)**

Do you think there is enough time for student physical activity at your school?

- Yes
- No
- Don't know
